# A Generation Shift in Mediterranean Diet Adherence and Its Association with Biological Markers and Health in Dalmatia, Croatia

**DOI:** 10.3390/nu13124564

**Published:** 2021-12-20

**Authors:** Jelena Šarac, Dubravka Havaš Auguštin, Mario Lovrić, Sarah Stryeck, Iva Šunić, Natalija Novokmet, Saša Missoni

**Affiliations:** 1 Centre for Applied Bioanthropology, Institute for Anthropological Research, 10000 Zagreb, Croatia; jsarac@inantro.hr (J.Š.); dhavas@inantro.hr (D.H.A.); iva.sunic@inantro.hr (I.Š.); 2 Institute for Anthropological Research, 10000 Zagreb, Croatia; natalija.novokmet@inantro.hr (N.N.); sasa.missoni@inantro.hr (S.M.); 3 Know-Center, Inffeldgasse 13, 8010 Graz, Austria; sarah.stryeck@tugraz.at; 4 Institute of Interactive Systems and Data Science, Graz University of Technology, Inffeldgasse 16c, 8010 Graz, Austria; 5 School of Medicine, “J. J. Strossmayer” University, 31000 Osijek, Croatia

**Keywords:** Mediterranean diet, MDSS score, Croatia, Eastern Adriatic islands, westernization, CRIBS cohort, NIH cohort, diet, lifestyle, biological markers

## Abstract

Previous studies have confirmed the beneficial effect of a Mediterranean diet in mitigating health issues related to cardiovascular disease, diabetes and obesity. However, rapid changes in the traditional way of life and the “westernization” of the diet in Mediterranean populations, especially in younger generations, has led to progressive abandonment of healthy dietary patterns. In order to investigate the generation shift in dietary patterns and lifestyle habits in the Mediterranean part of Croatia, we compared two cohorts of 610 women (266 pregnant and 344 non-pregnant) from the same region, but from different age groups. The MDSS score was derived from food frequency questionnaires. The results showed that the young, reproductively active generation (pregnant women) in Dalmatia, Croatia, although having a higher education and socioeconomic status, exhibits a more adverse eating behaviour (lower adherence to the Mediterranean diet) and lifestyle (excessive smoking in pregnancy) than the older population from the same region. Lower MDSS scores across aggregated age groups in both cohorts showed significant association with higher blood lipid levels and higher smoking frequency. In conclusion, Mediterranean diet adherence is associated with biological markers (age, lipid profile) and lifestyle (smoking) in our study, with a more adverse trend observed in the younger generation.

## 1. Introduction

The term Mediterranean diet was coined nearly 50 years ago by Ancel Keys, as the way of living and eating observed in the countries around the Mediterranean Basin [1,2]. It has been characterized by a high intake of olive oil, fruit, nuts, vegetables, legumes and cereals, a moderate intake of fish, poultry and red wine, and a rather low intake of dairy (mostly in the form of cheese or yoghurt), red and processed meat, animal fat and sweets. Adherence to this type of dietary pattern has been linked to numerous health benefits and it can help mitigate the burden of cardiovascular disease, breast cancer, depression, colorectal cancer, diabetes, obesity, asthma, cognitive decline etc. [3,4,5,6,7]. However, social and economic changes have worldwide led to important modifications in food patterns in the last few decades [8,9]. Nowadays, due to the increasing urbanization, globalization of the agricultural market and the development of mass food culture centered around a “western diet”, the Mediterranean diet and other related healthy dietary patterns are being progressively abandoned [10]. Dietary energy intake has been steadily increasing and it significantly exceeds per capita daily energy requirements, leading to health issues and high health expenditure on a global level. Recent research from different Mediterranean countries also revealed that especially younger generations are shifting from the traditional way of life towards a ”westernization” of their diet [11,12]. This is particularly evident in urban areas, where people have adopted a dynamic and fast lifestyle. Higher adherence to healthy food consumption is associated with higher socioeconomic status (SES) across studies, while the younger generations in southern Europe exhibit medium to low adherence to the Mediterranean diet [13,14,15].

Research has also shown that relatively rural island communities, located in the Mediterranean part of Croatia, show a similar trend of abrupt and drastic changes in dietary habits and lifestyle due to the globalization and transition processes from the 1990s onwards [16,17]. These changes are characterized by an increased consumption of red meat, poultry, milk, dairy products, sugar, and industrial, processed products and a decreased consumption of fish, fruits, and vegetables, accompanied by a drastically reduced level of physical activity. Consequently, an increase in the overweight and obesity trend, as well as in the occurrence of metabolic syndrome, diabetes mellitus, and coronary heart diseases has been observed in the Croatian Mediterranean region.

Although a healthy diet and lifestyle should be a priority during pregnancy, birth cohort studies have indicated that the general maternal adherence to the Mediterranean diet in Europe is low and does not meet the recommendations. Additionally, a recent study investigating adherence of pregnant women to the Mediterranean diet in Dalmatia, Croatia observed a non-Mediterranean dietary pattern and more pronounced adverse socioeconomic and lifestyle conditions in the island populations when compared to the urban mainland counterpart [17].

In order to investigate the generation shift in dietary patterns and lifestyle habits in Dalmatia, Croatia, we compared two cohorts (pregnant vs. non-pregnant women) from the same region, but from different age groups. Cohort studies are important in health sciences, since they can detect any change in health related to the possible risk factors that they have identified. Prospective, longitudinal studies are especially useful and both cohorts used in this study include several participant follow-ups. They have both been characterized in previous publications [17,18,19,20]. The aim of the study was to assess the Mediterranean diet adherence in both cohorts using the Mediterranean Diet Serving Score (MDSS) and compare it to biological markers (age, biochemical profile, blood pressure, anthropometry), as well as socioeconomic (education level, income) and lifestyle variables (smoking, physical activity). The main study hypothesis is that we witness a generation shift in dietary habits and lifestyle in Dalmatia, Croatia and that pregnancy does not encourage women to change their behavior towards healthier life choices.

## 2. Materials and Methods

Data generated in two cohorts (the CRIBS and the NIH cohort) were utilized in this study. The CRIBS cohort represents 266 pregnant women (19-46 years of age) from Dalmatia, Croatia and the NIH cohort encompasses 344 women (22–91 years of age) from the same region. Demographic, socioeconomic, and lifestyle data were collected through self-completed questionnaires in both cohorts. Medical data were retrieved from the hospital medical records in the CRIBS cohort and through questionnaires filled out by a medical professional in the NIH study. Anthropometric measurements were taken by trained medical staff in both cohorts. Blood samples from pregnant CRIBS women were taken in the second trimester (between 22nd and 26th week of gestation) and from NIH participants during fieldwork. All the biochemical analyses were performed at the accredited laboratory of the Dubrava Clinical Hospital in Zagreb, Croatia. All data from the CRIBS cohort are pregnancy-adjusted. More detailed descriptions of collected anthropometric, clinical and laboratory data from both cohorts are summarized in previous studies [18,19,20,21].

In this study, the following covariates were considered: age, smoking status (smoker, non-smoker, ex-smoker), physical activity at work and recreationally (high, moderate, low), parity, miscarriages, SES (education level, financial status), weight, height, systolic and diastolic blood pressure and biochemical parameters (glucose and diet-related blood lipid levels: triglycerides, cholesterol, HDL cholesterol, LDL cholesterol). Nutritional data in the CRIBS cohort were derived from the Dietary Adequacy Assessment Questionnaire for Adults (DAAQA), a food frequency questionnaire adapted from the Harvard Semiquantitative Food Frequency Questionnaire, as described in Havaš Auguštin et al. (2020) [17]. Dietary habits in the NIH cohort were assessed by a quantitative food frequency questionnaire (FFQ) used in several nutritional surveys of the Hvar population and tested for reproducibility and validity [22,23]. A Mediterranean Diet Serving Score was calculated for both cohorts as described in Monteagudo et al. 2015 [24]. The maximum possible MDSS score in the original study was 24 points, and the cut-off of ≥13.5 points was considered as good compliance. However, the maximum possible MDSS score in this study was 23 points and the cut-off was set at ≥12.5 points—we excluded one category (alcoholic beverages) from the calculation, since CRIBS is a cohort of pregnant women.

The data processing procedures and analyses were written in Python 3.7 (https://www.python.org/. Last accessed on 26 September 2021). Raw data preprocessing methods were conducted according to our previous work [17]—all frequency data were converted to daily frequencies. The final data set combined of the two cohorts consisted of 610 participants (266 from the CRIBS and 344 from the NIH cohort). For investigating the participants’ nutrition and lifestyle patterns, a generalized least squares models (GLS) with the statsmodels library were utilized [25]. The tested outcomes were the MDSS score and the cohort association (CRIBS or NIH). The two dataset groups were compared with all variables listed in Appendix A. Furthermore, the principal component analysis (PCA) was used to reveal potential differences in nutritional patterns between the two cohorts [26]. Prior to PCA, low variance and highly intercorrelated variables were removed and sub-selected from a list of variables, as well as scaled.

## 3. Results

### 3.1. Differences between the Cohorts

**Figure 1:**
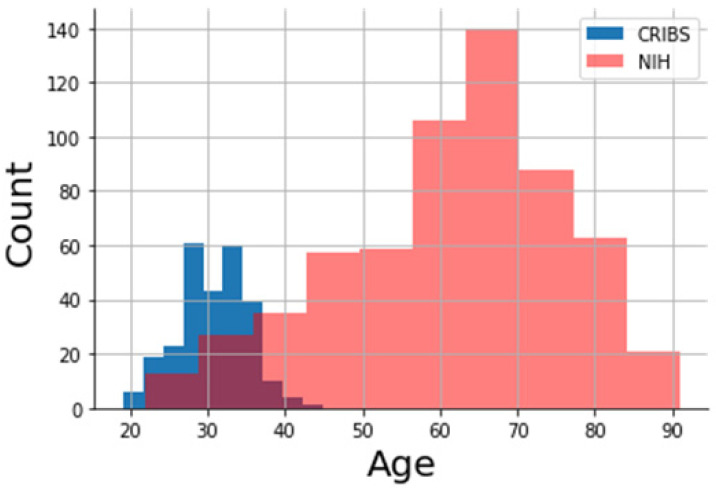
Age distribution of the two cohorts. The CRIBS cohort (blue colored histogram) consists of 266 pregnant women, while the NIH cohort consists of 344 non-pregnant women (pink colored histogram).

Prior to the assessment of relationships and patterns between the two cohorts, we analyzed general cohort descriptors and differences. Figure 1 shows age distributions across both cohorts. The NIH cohort is larger (*N* = 344) compared to the CRIBS (*N* = 266), with NIH peeking around a median of 62 years and CRIBS with a median at 31 years of age. The union of the two cohorts has a median age of 41 years.

Table 1 shows the baseline characteristics of the study participants and cohort differences in age distribution, women’s lifestyle and socioeconomic status (smoking, level of education, employment, financial status of the family), and MDSS. Indicators of socioeconomic status (level of education, proportion of employed women and financial status) are all significantly higher in the group of women from the CRIBS cohort, whereas age and MDSS are significantly higher in the NIH cohort. No significant difference between smoking prevalence has been detected between the two cohorts.

**Figure 2:**
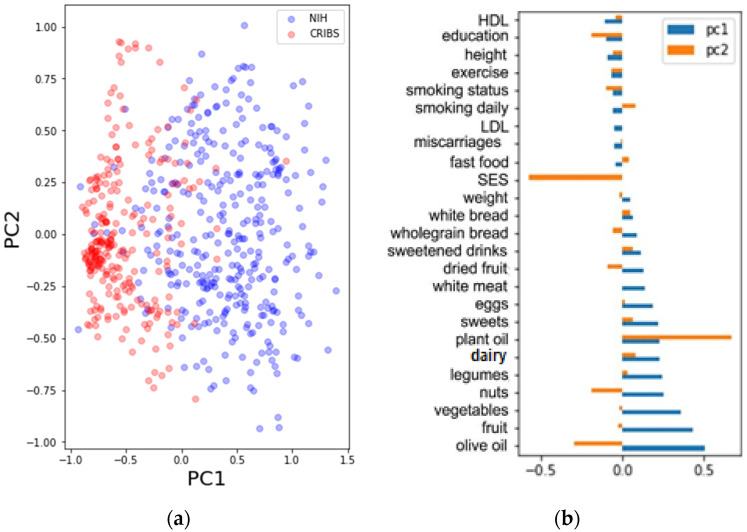
(**a**) PCA scores of the nutrition variables (Appendix A). The scores are colored based on the cohort (blue = NIH, red = CRIBS); (**b**) A bar plot of the PCA loadings.

The differences between the two groups were also assessed by the utilization of PCA. PCA was fed with 25 variables after the removal of variables with low-variance (<2%) and intercorrelated variables (>0.80). The variables are given in Appendix A. Since PCA is an unsupervised analysis, we made sure no variables which could be driving the split between the variables are present (such as age—annotated in Appendix A). We generated a PCA scores plot with two principal components, which is given in Figure 2a, while the respective loadings are shown in Figure 2b. We colored the PCA score based on the cohort association (blue for NIH, red for CRIBS). The scores plot showed a good separation between the cohorts based on PC1.

The loading plot (Figure 2b) shows which variables have a high loading on the separation between the two groups in the scores plot in Figure 2a. They are colored with respect to the cohort association (red = CRIBS, blue = NIH) on the first principal component. The highest loadings are assigned to the frequencies of consuming olive oil, fruits, vegetables, nuts and legumes (the first five). The variables used for PCA were utilized in a GLS model to assess differences between the cohorts (0 = CRIBS, 1 = NIH). All coefficients from the generated model including 25 variables were inspected (Appendix A). Results given in Table 2 show that five variables have coefficients with a high statistical significance (*p* < 0.001). The coefficients are sorted in descending order in the table. Positive trends towards the NIH cohort are therefore the frequencies of eating vegetables, white meat, sweets, fruits and dairy products. Negative trends towards the NIH cohort are the level of education, frequency of eating dried fruits, height, number of miscarriages, smoking status, HDL and smoking frequency. The age variable was not considered in this calculation due to it being a strong discriminator between the cohorts.

### 3.2. Adherence to Mediterranean Diet Associates with Increasing Age

**Figure 3:**
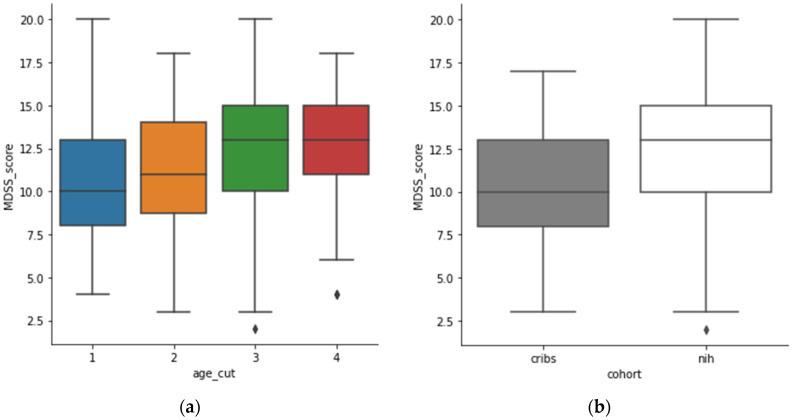
(**a**): MDSS score across age categories (0–33 = blue, 31–46 = orange, 46–65 = green, 65–91 = red); (**b**): MDSS score across the two cohorts (CRIBS = grey, NIH = white).

We aggregated the age variable of both cohorts together into four groups: “19–31” (*N* = 166), “32–46” (*N* = 164), “47–65” (*N* = 141) and “65–91” (*N* = 139). An inspection of the MDSS scores across aggregated age showed an increasing trend of Mediterranean diet adherence with increasing age (Figure 3a). The median MDSS score was ascending with the age groups in the following order: “65–91” ~ “46–65” > “31–46” > “19–31”. Therefore, the two older age groups have a similar adherence to the Mediterranean diet. Furthermore, we inspected the adherence of participants in both cohorts (Figure 2b) and we observed that the NIH cohort had a higher adherence compared to the CRIBS cohort (encompassing only pregnant women).

The relationship between age and the MDSS (outcome) was further tested in a GLS model. The model was fed two predictive variables: age cut (four categories) and cohort (0 = CRIBS, 1 = NIH). Both variables showed positive coefficients with regards to the outcome (MDSS) for age of 1.0820 (stderr = 0.559; *p* = 0.053) and cohort of 1.5590 (stderr = 0.419; *p* = 0.000). The results indicated that the MDSS score increased around +1 for age categories and, for the cohort, the increase was ~1.5 from CRIBS to NIH. The variables were corrected for each other.

### 3.3. Lifestyle Is Associated with Mediterranean Dietary Pattern

The comparison of adherence to the Mediterranean diet in a total sample of participants from both cohorts with different biological variables and lifestyle characteristics is presented in Table 3. Both cohorts were analyzed using the Mediterranean Diet Serving Score (Monteagudo et al. 2015) and the cut-off was set at 12.5 points (<12.5 points for low adherence, ≥12.5 points for high adherence). We inspected the association of the calculated MDSS in both cohorts with education, income, working status, physical activity, smoking and alcohol consumption, as well as with age, anthropometry, blood pressure and biochemical profile.

Smoking prevalence was significantly higher in the group of women who do not follow the recommendations of the Mediterranean diet (*p* < 0.001). Although BMI was on the borderline of normal weight and overweight in both groups, the results showed significantly higher scores in the group with higher MDSS (*p* < 0.01). Significant differences were observed for fasting glucose and lipids levels. Although in a normal range for both groups of participants, significantly lower levels of blood glucose were found in the non-adhering group of women (*p* < 0.001). Also, all parameters of serum lipids levels (triglycerides, total cholesterol, HDL and LDL) were significantly lower in the group with higher adherence to the Mediterranean diet. Higher blood pressure (*p* < 0.001 for diastolic and *p* < 0.01 for systolic blood pressure), glucose levels (*p* < 0.001) and BMI values (*p* < 0.01) have been observed in the adhering group.

In addition to the univariate statistical test, we have trained a GLS model on the MDSS score (binary, 1 if MDSS ≥ 12.5) which returned only four variables to have coefficients with a *p*-value below 0.01, namely “olive oil” (coef. 0.309 [0.208; 0.410]), “fruit” (coef. 0.268 [0.084; 0.452]); “plant oil” (coef. 0.150 [0.041; 0.259]) and “smoking daily” (coef. −0.346 [−0.586; −0.105]). The number of variables is expected, since variables are corrected for each other (as opposite to univariate tests). Even though MDSS consists of scoring for the given variables, we wanted to inspect which of these variables are driving the difference in MDSS the most. The model results show that a higher MDSS score is associated with using olive and plant oil, and eating fruits more frequently, whereas smoking is associated with low MDSS scores.

## 4. Discussion

We have compared two cohorts from the same Mediterranean region of Dalmatia, Croatia to investigate their diet, lifestyle habits and health and analyzed observed similarities and differences. The NIH cohort encompasses older women mainly from rural communities and as such, should reflect a more traditional diet and lifestyle. However, the traditional diet (home-cooked, unprocessed food from home-grown fruits and vegetables, dairy products from free-range goats and sheep) has become less present even in small, rural communities in Croatia, such as island populations. The observed changes are characterized by an increased consumption of red meat, sugar, and industrial, processed products and a sedentary lifestyle [16]. The NIH cohort has been analyzed previously with regards to the metabolic syndrome. A high prevalence of metabolic abnormalities (especially central obesity, dyslipidemia, and pro-inflammatory factors) has been observed in the sampled population [18,19]. The CRIBS cohort is represented by a younger generation and encompasses only pregnant women. Although healthy food patterns and lifestyle should be priorities during pregnancy and good or moderate adherence to the Mediterranean diet was expected in the CRIBS cohort, our previous study showed a poor level of adherence [17].

The main finding of the present study is that generational differences have been detected when comparing these two cohorts. Although the NIH cohort is older and has previously been characterized as a population at substantial risk for cardiovascular diseases, it still practices a healthier and more traditional lifestyle and diet when compared to the younger generation of pregnant women from the same region. Education level, employment status and financial status are significantly higher in the group of women from the CRIBS cohort. This finding reflects the change in modern society and generational differences where older women were more prone to stay at home and attend to household chores, while younger generations of women are more career-oriented. It should be emphasized that half of the women willing to be enrolled in the CRIBS study had a high educational level (college degree, master or PhD). However, despite being more educated and wealthier, younger women from Dalmatia adhere less to the Mediterranean diet in comparison to older women from the same geographical area. When inspecting the association between diet and participant age, higher age was directly associated with high adherence to the Mediterranean diet, similar to the other research conducted in the Mediterranean region which has also recognized the trend of older age groups being more prone to the traditional dietary habits than young adults [11,27,28]. When the two cohorts are analyzed in more detail, specific differences are observed. Namely, women from the NIH cohort eat more individual food items characteristic of the Mediterranean diet (vegetables, white meat, fruit, dairy), while pregnant women from the CRIBS cohort have an increased miscarriage rate and smoke more frequently. Although European statistics show that <10% of women continue to smoke during pregnancy in studies conducted after 2010, pregnant women in our birth cohort show a high prevalence (23%) [29]. This prevalence corresponds to the prevalence in the NIH cohort, indicating that no change in behavior occurs with pregnancy in Mediterranean Croatia. Maternal smoking is also usually a marker of social inequality, with higher rates of pregnant smokers observed among women with lower educational levels, lower income and unemployment [29]. However, most of the pregnant participants (in the CRIBS cohort) have a beneficial SES. Smoking, especially during pregnancy, is also directly associated with being unaware of the importance of healthy eating habits. From a subsample of smokers in the cohort of pregnant women, only 15% of them also follow a traditional healthy food pattern. In the NIH cohort the situation is a little bit better—42% of women who smoke adhere to the Mediterranean diet. Similar findings were confirmed in other investigated populations where smokers were less likely to comply with the Mediterranean diet and negative lifestyle behaviors were similarly reflected in unhealthy dietary patterns [28]. Physical activity levels and biochemical profiles were not significantly different between the cohorts.

When inspected in more detail, our study suggests that high olive and plant oil consummation, as well as eating fruit, are most responsible for a high MDSS score in our total sample. Certain lifestyle characteristics have also been associated with a healthy diet. Namely, smoking prevalence was significantly higher in the group of women who do not follow the recommendations of the Mediterranean diet. Previous studies also indicated that the Mediterranean diet shows significant beneficial effects regarding blood lipid control [30,31]. Our study confirmed this finding, since high adherence correlated with lower LDL levels, as well as lower triglycerides and cholesterol values when the whole sample is taken into consideration. Interestingly, higher blood pressure, glucose and BMI values, as well as lower HDL levels, have been observed in the adhering group. Although a healthy diet is usually associated with beneficial effects for these biological markers and body mass, obtained results can be explained by the higher age of participants, which then correlates with higher blood pressure and glucose values, as well as increased weight.

## 5. Conclusions

Urbanization, population growth and the progressive globalization of the food market have been identified as potential causative factors for the global shift in dietary and lifestyle habits and, particularly, the abandonment of the Mediterranean dietary pattern, which has significant repercussions on individual health. A generation shift has been observed in this study and it reflects itself in both nutritional habits (adherence to the Mediterranean diet) and lifestyle differences (smoking, socioeconomic status) between the two tested cohorts of women. Our study suggests that the young, reproductively active generation in Dalmatia, Croatia, although having a higher socioeconomic status and being pregnant, exhibits a more adverse eating behavior (lower Mediterranean Diet Serving Score) and lifestyle (excessive smoking in pregnancy) than the older population from the same region. Therefore, it is important to raise awareness among youth and encourage them to adopt healthy and sustainable lifestyle patterns based on balanced nutrition.

## Figures and Tables

**Table 1:** Baseline characteristics of participants by cohort.

Variable	Category	CRIBS N (%)	NIH N (%)	*p* Value
age	19–31	151 (56.8%)	14 (4%)	<0.001
	32–46	115 (43.2%)	49 (14.2%)	
	47–64	0 (0%)	128 (37.2%)	
	65–91	0 (0%)	153 (44.5%)	
smoking status	smokers	62 (23.3%)	84 (24.4%)	>0.05
	non-smokers	204 (76.7%)	260 (75.6%)	
education	high	125 (47%)	65 (18.9%)	<0.001
	low	141 (53%)	279 (81.1%)	
employment status	employed	209 (78.6%)	112 (32.%)	<0.001
	unemployed	57 (21.4%)	232 (67.4%)	
financial status	high	78 (29.3%)	69 (20.1%)	<0.01
	medium	169 (63.5%)	241 (70%)	
	low	19 (7.1%)	34 (9.9%)	
MDSS score	high	74 (27.8%)	184 (53.5%)	<0.001
	low	192 (72.2%)	160 (46.5%)	

**Table 2:** Coefficients of variables used in PCA. The PCA plots are given in Figure 2.

Variable	Coefficient	*p*-Value
vegetables	0.736	0.000
smoking status	−0.359	0.000
HDL	−0.39	0.000
smoking daily	−0.482	0.000
sweets	0.251	0.000
white meat	0.297	0.001
education	−0.164	0.001
fruit	0.157	0.001
dairy	0.125	0.006
height	−0.183	0.006
miscarriages	−0.186	0.006
dried fruit	−0.18	0.008

**Table 3:** Association of biological and lifestyle characteristics with the Mediterranean Diet Serving Score.

Variable	Low MDSS	High MDSS	*p*-Value
smoking	6.81	3.644	<0.001
diastolic blood pressure	71.026	75.081	<0.001
glucose	5.091	5.521	<0.001
systolic blood pressure	121.971	126.586	<0.01
HDL	1.756	1.668	<0.01
BMI	24.084	25.26	<0.01
triglycerides	1.705	1.566	<0.01
total cholesterol	6.433	6.121	<0.01
LDL	3.93	3.783	<0.05

## Data Availability

Data are not publicly available. Data can be made available upon request.

## Appendix A

Not all variables are included in all analyses, as presented in the table below.

**Table A1:** A list of variables per analysis.

Variable Short Name	Variable Description	Included in Model	Excluded in Stat	Excluced in PCA	Exlcuded in GLS
dairy	daily consummation of dairy				
systolic blood pressure	systolic blood pressure value			x	x
other fat, oil	daily consummation of oil (excl. olive and plant oil)			x	x
dried fruit	daily consummation of dried fruit				
vegetables	daily consummation of vegetables				
wholegrain bread	daily consummation of wholegrain bread				
equipment	measure of socieconomic status according to equipment in the household				
total cholesterol	total cholesterol value			x	x
fast food	daily consummation of fast food				
potatoes	daily consummation of potatoes			x	x
height	height				
financial status	monthly household income			x	x
pasta, rice	daily consummation of pasta and rice			x	x
red meat	daily consummation of red meat			x	x
eggs	daily consummation of eggs				
age	age			x	
sweetened drinks	daily consummation of sweetened drinks				
glucose	fasting glucose value			x	x
nuts	daily consummation of nuts				
number pregnancy	number of pregnancies or parity			x	x
diastolic blood pressure	diastolic blood pressure value			x	x
fruit	daily consummation of fruit				
MDSS 2 kat	Mediterranean Diet Serving Score in two categories (low and high)		x	x	x
white meat	daily consummation of white meat				
smoking years	years of smoking			x	x
miscarriages	number of miscarriages				
HDL	HDL cholesterol value				
smoking daily	daily number of cigarettes				
cohort	NIH and CRIBS cohort		x	x	x
fish	daily consummation of fish			x	x
white bread	daily consummation of white bread				
MDSS score	Mediterranean Diet Serving Score in numbers (12.5 cut-off)		x	x	x
BMI	body mass index			x	x
sweets	daily consummation of sweets				
olive oil	daily consummation of olive oil				
exercise	daily activity level in three categories (low, moderate, high)				
sex	sex		x	x	x
legumes	daily consummation of legumes				
triglycerides	triglyceride value			x	x
LDL	LDL cholesterol value				
smoking status	smoking status in three categories (smoker, non-smoker, ex-smoker)				
location	mainland vs. islands		x	x	x
plant oil	daily consummation of plant oil				
working status	working/employment status			x	x
education	education level				
weight	weight				
